# Supplementary material for: Elovanoids Counteract Inflammatory Signaling, Autophagy, Endoplasmic Reticulum Stress, and Senescence Gene Programming in Human Nasal Epithelial Cells Exposed to Allergens
Source: Pharmaceutics. 2022 Jan 4;14(1):113. doi: 10.3390/pharmaceutics14010113 (PMC8778361; doi:10.3390/pharmaceutics14010113)
Supplement: Supplementary file 1 [file pharmaceutics-14-00113-s001.zip › pharmaceutics-1453084-supplementary.pdf]

pharmaceutics

MDPI

Supplementary Materials: Elovanoids Counteract Inflammatory Signaling, Autophagy, Endoplasmic Reticulum Stress, and Senescence Gene Programming in Human Nasal Epithelial Cells Exposed to Allergens

Alfredo Resano, Surjyadipta Bhattacharjee, Miguel Barajas, Khanh V. Do, Roberto Aguado-Jiménez, David Rodríguez, Ricardo Palacios and Nicolás G. Bazán

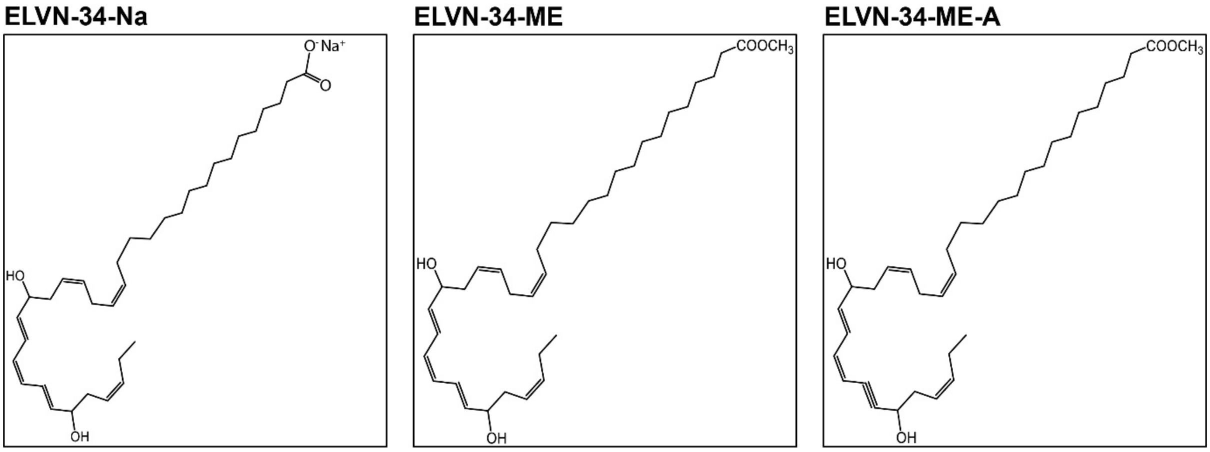

Figure S1. Structures of Elovanoids (ELV). ELVN-34:6 Na, ELVN-34:6 Me, and ELVN-34:6 Me-A at (500 nM) were used for experiments in this study.

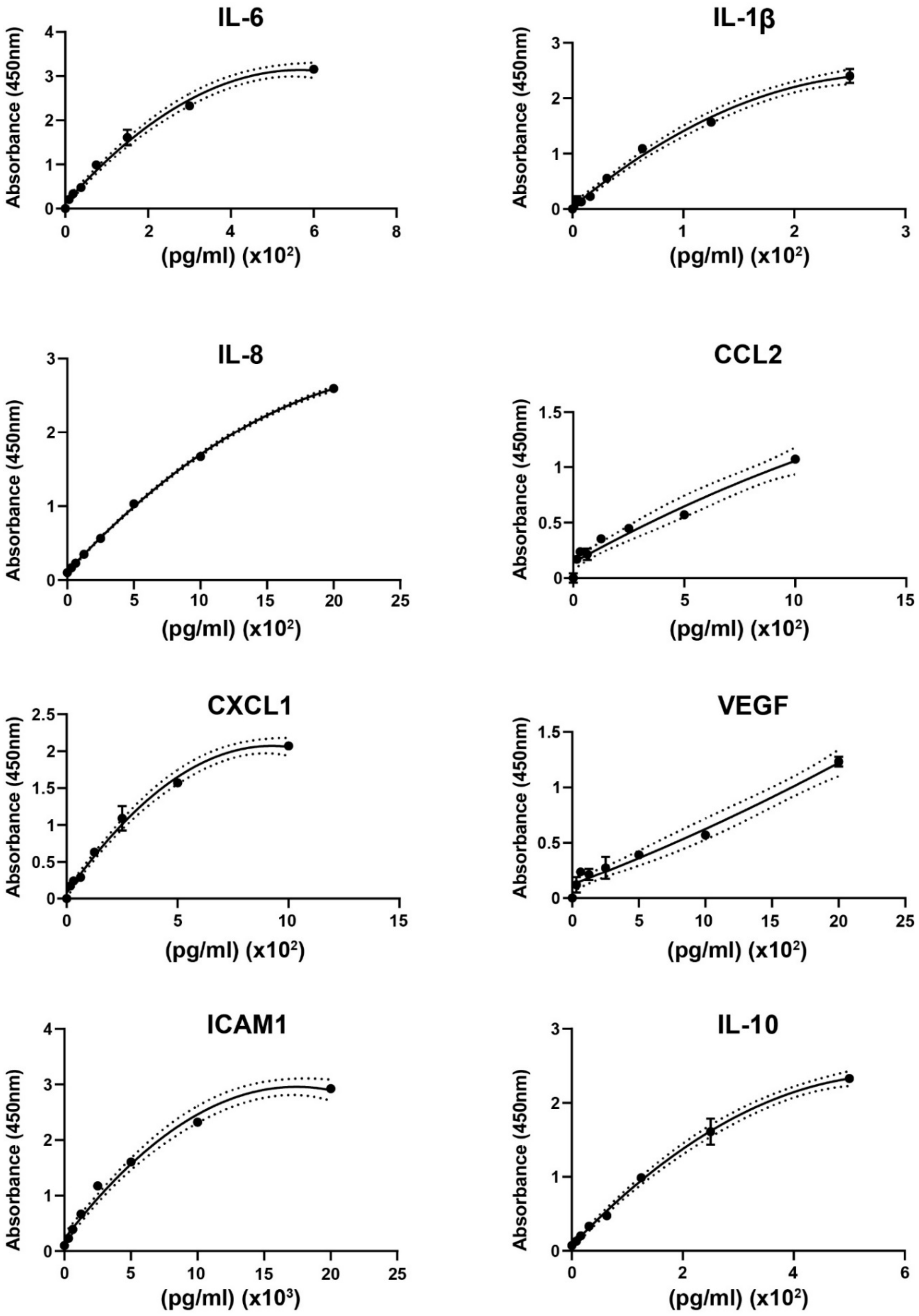

Figure S2. ELISA Standard curves. For the ELISA assays mentioned in this study, the standard curves for each assay are given above.

Table S1. List of primers used in the study.

Protein	Gene Name	Forward Sequence	Reverse Sequence
ATG3	*Atg3*	GATGGCGGATGGGTAGATACA	TCTTCACATAGTGCTGAGCAATC
ATG5	*Atg5*	AGAAGCTGTTTCGTCCTGTGG	AGGTGTTTCCAACATTGGCTC
ATG7	*Atg7*	ATGATCCCTGTAACTTAGCCCA	CACGGAAGCAAACAACTTCAAC
Beclin-1	*Becn1*	GGTGTCTCTCGCAGATTCATC	TCAGTCTTCGGCTGAGGTTCT
p62	*Nup62*	CCTTCCAACCAGCCACAAGTAC	CGAAGCAAGAGTCGCTGTTCCA
ATF6	*Atf6*	GACTCACCCATCCGAGTTGTG	CTCCCAGTCTTCATCTGGTCC
CHOP	*Aff4*	CTGGAAGCCTGGTATGAGGAT	CAGGGTCAAGAGTAGTGAAGGT
IRE1	*Ern1*	CCGAACGTGATCCGCTACTTCT	CGCAAAGTCCTTCTGCTCCACA
MMP8	*Mmp8*	AATCCTTGCCCATGCCTTTCAACC	CCAAATTCATGAGCAGCCACGAGA
MMP2	*Mmp2*	CCTTAAAAGTATGGAGCGACGTCA	AGCGTTCCCATACTTTACGCG
MMP9	*Mmp9*	ACGACATAGACGGCATCCAGTATC	AGGTATAGTGGGACACATAGTGGG
MMP3	*Mmp3*	ACCAACCTATTCCTGGTTGCTGCT	ATGGAAACGGGACAAGTCTGTGGA
MMP12	*Mmp12*	TAGAAGCAACTGGGCAACTGGACA	ACCGCTTCATCCATCTTGACCTCT
TIMP1	*Tipm1*	GGAGAGTGTCTGCGGATACTTC	GCAGGTAGTGATGTGCAAGAGTC
TIMP2	*Tipm2*	ACCCTCTGTGACTTCATCGTGC	GGAGATGTAGCACGGGATCATG
$p21^{CIP}$	*Cdkn1a*	TGTCCGTCAGAACCCATGC	AAAGTCGAAGTTCCATCGCTC
$p16^{INK4a}$	*Cdkn2a*	GGGGGCACCAGAGGCAGT	GGTTGTGGCGGGGGCAGTT
$p27^{KIP}$	*Cdkn1B*	TAATTGGGGCTCCGGCTAACT	TGCAGGTCGCTTCCTTATTCC
p53	*Trp53*	CCCCTCCTGGCCCCTGTCATCTTC	GCAGCGCCTCACAACCTCCGTCAT
IL-1α	*Il-1α*	TGTATGTGACTGCCCAAGATGAAG	AGAGGAGGTTGGTCTCACTACC
IL-6	*Il-6*	CTGCAAGAGACTTCCATCCAG	AGTGGTATAGACAGGTCTGTTGG
IL-1β	*Il-1β*	AGCTACGAATCTCCGACCAC	CGTTATCCCATGTGTCGAAGAA
ACTB	*ActB*	AGCCTCGCCTTTGCCGA	CTGGTGCCTGGGGCG
GAPDH	*Gapdh*	TGGACCTGACCTGCCGTCTA	CCCTGTTGCTGTAGCCAAATTC
